# The Algal Antioxidant Carotenoid Diatoxanthin as a Modulator of Inflammation and Angiogenesis in Triple-Negative Breast Cancer Cells

**DOI:** 10.3390/antiox15020205

**Published:** 2026-02-04

**Authors:** Danilo Morelli, Luana Calabrone, Luisa Di Paola, Giovanna Chiorino, Paola Ostano, Douglas M. Noonan, Giovanni Corso, Adriana Albini

**Affiliations:** 1 Fondazione IRCCS Istituto Tumori di Milano, 20133 Milan, Italy; d.morelli6@campus.unimib.it; 2 ISB—Ion Source & Biotechnologies Srl, 20017 Rho, Italy; luana.calabrone@gmail.com; 3 Unit of Chemical-Physics Fundamentals in Chemical Engineering, Department of Science and Technology, Università Campus Bio-Medico, 00128 Rome, Italy; l.dipaola@unicampus.it; 4 Lab of Cancer Genomics, Fondazione Edo ed Elvo Tempia, 13900 Biella, Italy; giovanna.chiorino@fondazionetempia.org (G.C.); paola.ostano@fondazionetempia.org (P.O.); 5 Department of Biotechnology and Life Sciences, University of Insubria, 21100 Varese, Italy; douglas.noonan@gmail.com; 6 Unit of Molecular Pathology, Biochemistry and Immunology, Istituto di Ricovero e Cura a Carattere Scientifico (IRCCS), MultiMedica, 20138 Milan, Italy; 7 Division of Breast Surgery, European Institute of Oncology (IEO), Istituto di Ricovero e Cura a Carattere Scientifico (IRCCS), 20141 Milan, Italy; giovanni.corso@ieo.it; 8 Department of Oncology and Hematology, University of Milan, 20122 Milan, Italy; 9 Scientific Directorate, European Institute of Oncology (IEO), Istituto di Ricovero e Cura a Carattere Scientifico (IRCCS), 20141 Milan, Italy

**Keywords:** diatoms, diatoxanthin, triple-negative breast cancer, angiogenesis, antioxidants, inflammation, ferroptosis, chemotherapy, RNA-Seq

## Abstract

Algal carotenoids play a promising role in handling chronic diseases due to their diverse bioactive properties, including anti-inflammatory, antioxidant, and anticancer effects. This study assesses the activity of the antioxidant xanthophyll diatoxanthin (Dt), derived from marine diatoms, against triple-negative breast cancer (TNBC) cells using in vitro models, gene expression evaluation, and explores its role in potentiating the cytotoxic effect of chemotherapy. Dt exhibited selective activity against MDA-MB-231 and BT-549 TNBC cells at concentrations ≥12.5 ng/mL, with maximal effects observed at 25 ng/mL while sparing human umbilical vein endothelial cells (HUVECs) at these doses. When combined with doxorubicin (0.1–0.5 μM), Dt enhanced the anti-tumor efficacy in both TNBC cell lines, further reducing cell viability compared with doxorubicin alone (*p* < 0.05–0.001). Dt also exerted its activity in inhibiting migration and chemotaxis by approximately 30–50% compared with the controls (*p* < 0.01) and suppressing 3D-tumor spheroid growth at day 12 (up to >50% reduction, *p* < 0.001). Notably, secretome analysis revealed Dt-induced changes in inflammatory, oxidative and angiogenic mediators, highlighting its ability to modulate the TNBC microenvironment. Dt also downregulated key pro-survival, pro-angiogenic and pro-tumorigenic genes in both TNBC cell lines, supporting its role in disrupting oncogenic pathways. Angiogenesis-related genes were significantly reduced. Dt also decreased the expression of angiogenic mediators in HUVECs, supporting Dt’s role in inhibiting tumor vascularization. Results on gene expression regulation were also confirmed by RNA-Seq analysis. These findings pose Dt as a promising chemopreventing candidate in the challenging fight against TNBC, a well-known type of cancer that is aggressive and resistant to conventional therapies, targeting critical pathways for tumor survival, such as inflammation, angiogenesis, tumor cell growth, and cell migration. Given its selective activity against TNBC cells, ability to enhance chemotherapy efficacy, and modulation of the tumor microenvironment, Dt holds promise as a complementary drug for cancer prevention and interception. Future studies should focus on validating these effects in vivo and exploring Dt’s potential in combinatorial treatment strategies for cancer.

## 1. Introduction

Natural compounds play a crucial role in disease treatment due to their diverse bioactive properties, including anti-inflammatory, antioxidant, and anticancer effects [1,2]. Their ability to modulate key biological pathways makes them valuable candidates for both conventional and adjunctive treatments [3]. In the cancer context, natural compounds are increasingly recognized for their role in promoting apoptosis, inhibiting proliferation, suppressing angiogenesis, altering cancer cell morphology, and modulating immune function [4], including the induction of immunogenic cell death, a regulated form of tumor cell death [5]. Carotenoids, the most abundant natural pigments, are produced by photosynthetic organisms and some archaea, fungi, algae and animals [6] and are classified as oxygen-free carotenes or oxygen-containing xanthophylls [7]. The reactive oxygen species (ROS)-scavenging ability of dietary carotenoids may help lower the risk of cancer and other diseases [8]. Specifically, xanthophylls have demonstrated diverse bioactive properties, including antioxidant, anti-inflammatory, and anti-angiogenic effects [9]. Among the xanthophylls, fucoxanthin, which is present in the macroalgae and microalgae, has also been widely studied for its chemopreventive potential in our lab [10,11] and has paved the way for investigating similar molecules. In the scenario of marine compounds, diatoms are emerging as promising bio-platforms for producing bioactive molecules for biomedical applications [5,12], including cancer prevention and interception [13]. Diatoms produce the xanthophyll diatoxanthin (Dt), a pigment that plays an antioxidant role, protecting against high light damage [14,15,16], nutrient depletion [17], and viral attacks [18]. In the context of SARS-CoV-2 infection, Dt has shown its potential as a novel therapeutic candidate for the treatment and/or prevention of severe inflammatory syndromes [19]. Additionally, Dt regulates iron metabolism [17], particularly through ferroptosis, a type of regulated cell death triggered by oxidative stress, nutrient deprivation, and other cellular stresses [17,20]. Dt also showed cytotoxic effect against breast [21] and prostate cancer cell lines [22]. Interestingly, among the several pigments tested, Dt showed the greatest ability to reduce metalloproteinase-9 (MMP-9) levels and significantly lowered interleukin (IL)-1β in melanoma cells [20]. To strengthen the knowledge about Dt chemopreventive action and its possible role in enhancing the effects of chemotherapy, we used cellular models of breast cancer (BC).

BC is the most prevalent cancer among women [23] and is a leading cause of cancer mortality worldwide [24]. Triple-negative breast cancer (TNBC), which displays the poorest prognosis and the highest mortality rate, represents 15% of BC cases. TNBCs are characterized by the lack of expression of estrogen receptor (ER), progesterone receptor (PR), and human epidermal growth factor receptor 2 (HER2), making them unresponsive to hormone-based and HER2-targeted preventive therapies [25,26]. A significant proportion of TNBC is associated with inherited mutations, especially BRCA1 mutations [27]. Treatment options for TNBC are limited [28] and primarily rely on chemotherapy agents, such as anthracyclines and taxanes, which may be used in combination with approved biological therapies [29,30,31,32]. However, the systemic toxicity of chemotherapy and the development of drug resistance [33] underscore the urgent need for alternative or adjunctive therapeutic strategies [34]. Only recently have PARP inhibitors and antibody drug conjugates surfaced as novel therapeutics [35], with anthracyclines still the favorite option. 

These characteristics make TNBC an optimal model for studying the potential chemopreventive and interception action of natural compounds, as well as their ability to enhance responsiveness to chemotherapy. In this context, the anthracycline, doxorubicin (Doxo), is one of the main reference chemotherapeutic agents because such regimens remain the backbone of systemic therapy for TNBC [36]. Its well-established mechanism of action and toxicity profile [37] make it an appropriate positive control and provide a clinically relevant benchmark for evaluating potential additive effects with other agents. Evaluating the combination of synthetic or natural agents with Doxo is essential to enhance therapeutic efficacy and potentially reduce dosing-related toxicities in TNBC patients. Natural antioxidant compounds like Dt may offer cancer preventive potential and anti-tumor effects by targeting alternative molecular pathways or enhancing the effects of chemotherapy, as demonstrated for other similar molecules [38], thus possibly mitigating its toxicity.

This study expands current knowledge on the effects of Dt on BC by employing two TNBC models. In addition to assessing cell viability, we investigated the effects of Dt on spheroid growth, angiogenesis, and inflammation, both as a standalone agent and combined with Doxo. Effects on endothelial cells were also examined. Modulation of gene expression by Dt has also been studied by RT PCR and further by RNA-Seq analysis.

## 2. Materials and Methods

### 2.1. Cell Culture and Reagents

Human TNBC cell lines, MDA-MB-231 and BT-549, were obtained from the American Type Culture Collection (ATCC) (Manassas, VA, USA), and human umbilical vein endothelial cells (HUVECs) from Lonza (Basel, Switzerland). MDA-MB-231 and BT-549 cells were cultured in Dulbecco’s modified Eagle medium (DMEM; Gibco, Thermo Fisher Scientific™, Waltham, MA, USA) and Roswell Park Memorial Institute (RPMI) 1640 medium (Gibco, Thermo Fisher Scientific™, Waltham, MA, USA), respectively, supplemented with 10% fetal bovine serum (FBS; Gibco, Thermo Fisher Scientific™, Waltham, MA, USA), 2 mM L-glutamine, and 1% penicillin/streptomycin (Pen/Strep; Sigma-Aldrich^®^, St. Louis, MO, USA). HUVECs were grown in endothelial cell basal medium (EBM™, Lonza) enriched with endothelial cell growth medium (EGM™ SingleQuots™, Lonza), 10% FBS, 2 mM L-glutamine, and 1% Pen/Strep. These cells were maintained at 37 °C in a humidified atmosphere containing 5% CO_2_ and regularly screened for mycoplasma contamination.

Freeze-dried pure Dt (C_40_H_54_O_2_, CAS No. 31063-73-7; Simplified Molecular Input Line Entry System (SMILES) notation: CC1=C(C(C[C@@H](C1)O)(C)C)/C=C/C(=C/C=C/C(=C/C=C/C=C(\C)/C=C/C=C(\C)/C#CC2=C(C[C@H](CC2(C)C)O)C)/C)/C; Figure S1) was obtained from Sigma-Aldrich^®^ (St. Louis, MO, USA) and subsequently dissolved in ethanol (EtOH). Doxo was obtained from Selleck Chemicals (Houston, TX, USA), dissolved in water, and stored as a 10 mg/mL stock solution.

### 2.2. Viability Assays

Cell viability was evaluated using the MTT (3-(4,5-dimethylthiazol-2-yl)-2,5-diphenyltetrazolium bromide) assay to determine dose-dependent effects on cellular proliferation [39]. HUVECs and TNBC cells (5 × 10^3^ cells/well) were seeded into 96-well plates and treated with Dt at various concentrations (6.25–100 ng/mL) for 24, 48 and 72 h. Cell media containing ethanol (EtOH) served as a vehicle control to confirm that observed effects were attributable to Dt and not the solvent. Untreated cells (UT) were used as negative controls. For TNBC cells, a subsequent experiment, which combined Dt (25 ng/mL) with Doxo at concentrations of 0.1, 0.2 and 0.5 μM, was carried out to evaluate additive effects. Doxo alone (0.05–1 μM) was also tested, with no vehicle control for Doxo as its solvent is water. After treatment, cells were incubated with MTT (10 μL; 5 mg/mL stock) for 3 h at 37 °C. Formazan crystals were dissolved with 100% DMSO, and absorbance was measured at 570 nm using a SpectraMax^®^ M2 spectrophotometer (Molecular Devices, San Jose, CA, USA). All experiments were performed in triplicate, with eight technical replicates per condition.

### 2.3. Generation of 3D-Tumor Spheroids

3D-tumor spheroids were generated using a hanging drop method to replicate a physiologically relevant tumor microenvironment [40]. MDA-MB-231 and BT-549 cells were cultured as 20 μL hanging drops at 4 × 10^3^ cells/drop in complete DMEM or RPMI medium, respectively. Spheroids were grown under five conditions: UT, culture medium supplemented with ethanol (control cells [Ctr]) as ethanol is the solvent of Dt, Doxo (0.5 μM), Dt (25 ng/mL), and Doxo + Dt. The EtOH concentration in the Dt treatments matched the solvent control. After spheroid formation (within 24 h), spheroids were transferred to 96-well plates (one spheroid/well) coated with 2% agar in fresh medium, with treatments refreshed every 48 h. Growth was monitored over 12 days, and images were acquired at days 3, 6, and 12. Spheroid area was measured using ImageJ software (Version 1.54) and normalized to day 3. Diameter was calculated from the area using d = √(4A/π). Only one Doxo concentration (0.5 μM) was used due to challenges in the 3D-culture technique. Necrotic regions, prominent in the Doxo and Doxo + Dt treatments, were excluded from the diameter calculations.

### 2.4. Boyden Chamber Assay

The migration ability of MDA-MB-231 and BT-549 TNBC cells was assessed using a modified Boyden chamber assay [41]. Cells were synchronized by overnight incubation in starvation DMEM or RPMI 1640 medium, then seeded (4 × 10^4^ cells/well) into the upper chamber with starvation medium containing Dt (25 ng/mL) or EtOH (Ctr). A fibronectin-coated (2 μg/mL) 10 μm pore-size polycarbonate filter separated the upper and lower chambers. The lower chamber contained DMEM or RPMI 1640 with 10% FBS to establish a chemoattractant gradient. Positive controls used serum-free medium in the upper chamber and complete medium in the lower chamber, while negative controls used serum-free medium in both chambers. After 6 h at 37 °C and 5% CO_2_, non-migrated cells were removed from the upper filter surface. The filter was washed, fixed in 4% PFA for 10 min at RT, stained with crystal violet, and imaged using a Zeiss™ Axio Observer A1 microscope (Oberkochen, Germany) with a digital camera at 10× magnification. Each experimental condition was repeated four times, with three fields per filter imaged and analyzed. Migration was quantified by averaging cell counts across fields and replicates. Image analysis was performed using ImageJ software (National Institutes of Health, Bethesda, MD, USA). The experiment was repeated three times.

### 2.5. Scratch Wound Healing Migration Assay

The anti-migratory effect of Dt (25 ng/mL) on the MDA-MB-231 and BT-549 TNBC cells was evaluated using a scratch wound assay [42]. Cells (5 × 10^4^/well) were seeded in 12-well plates and incubated at 37 °C with 5% CO_2_ until confluence. An ~1 mm-wide scratch was made using a micropipette tip, and monolayers were washed twice with PBS to remove debris. Cells were then incubated in 1% FBS medium containing Dt (25 ng/mL). Images of the scratch area were taken at t_0_ and after 24 h using a Leica™ DM IL LED inverted microscope (Leica Microsystem, Milan, Italy) with a digital camera at 4× magnification. Wound closure was assessed by measuring the uncovered area at both time points. To quantify migration, the uncovered and migrated cell-covered areas were color-coded in Adobe Photoshop^®^ (version 21.0.6) and analyzed with ImageJ software. The percentage of area covered by migrated cells after 24 h was calculated relative to the initial uncovered area at t_0_. Each experimental condition was repeated four times, with images of three fields acquired per well. The mean covered area for each condition was calculated across replicates in three independent experiments. Doxo or Doxo + Dt treatments were excluded from this assay due to rapid cell death under low-serum conditions (1% FBS).

### 2.6. RNA Extraction and Gene Expression Profiling by qPCR

Quantitative PCR assessed the expression of genes involved in inflammation, angiogenesis, and tumor progression. MDA-MB-231 and BT-549 TNBC cells (1 × 10^6^) were seeded onto p100 plates. Upon reaching 80–90% confluence, cells were treated with Dt (25 ng/mL) for 6 h. HUVECs (2.5 × 10^5^ cells/well) were seeded in 6-well plates, adhered, and treated with Dt (25 ng/mL) in fresh medium for 6 h. Ctr received a complete medium supplemented with the same concentration of ethanol that is present in the solvent of the Dt dose used for treatment. UT received complete medium. Total RNA was extracted following standard protocols. RNA was isolated using the TRIzol method, and RNA concentration was determined with a Nanodrop™ Spectrophotometer (Thermo Fisher Scientific, Waltham, MA, USA). cDNA was synthesized using 1000 ng of RNA and the SuperScript™ VILO™ cDNA Synthesis Kit (Thermo Fisher Scientific, Waltham, MA, USA). qPCR was performed with SYBR Green Master Mix (Applied Biosystems, Waltham, MA, USA) on a QuantStudio™ 6 Flex System (Applied Biosystems, Waltham, MA, USA), with reactions conducted in triplicate and repeated three times. Primers were designed using NCBI Primer-Basic Local Alignment Search Tool (Primer BLAST, https://www.ncbi.nlm.nih.gov/tools/primer-blast/index.cgi accessed on 25 November 2025; details in Supplementary Table S1) and purchased from IDT (Coralville, IA, USA). Data collected over 40 cycles were analyzed with the MultiExperiment Viewer Software (MeV software, https://github.com/web-mev/ accessed on 25 November 2025).

### 2.7. Secretome Analysis

The proteomic profile of the secretome from the HUVECs, MDA-MB-231 and BT-459 cells was quantitatively analyzed using the RayBio™ Human Cytokine Antibody Array 3 and RayBiotech^®^ Human Angiogenesis Antibody Array (RayBiotech Life, Peachtree Corners, GA, USA) [43]. In brief, the antibody array membranes were first incubated with blocking buffer for 1 h, followed by an overnight incubation at 4 °C with 1 mL of secretome from the different cell lines (2000 µg/mL). After thorough washing, the membranes were treated with a biotinylated antibody cocktail for 2 h at room temperature under constant shaking. This was followed by a 2-h incubation with HRP-streptavidin at room temperature. The membranes were then developed using the chemiluminescent detection reagent provided in the kit, acquiring images after different periods of exposure.

### 2.8. RNA-Seq

Cells were treated with different concentrations of Dt and treatment was run on replicates. HUVECs were previously stimulated with tumor necrosis factor-α (TNF-α) to simulate inflammatory situation. Total RNA (totRNA) was isolated from treated cells and UT using the miRNeasy Mini Kit (Qiagen, Hilden, Germany) according to the manufacturer’s protocol. DNase treatment was carried out with the Turbo DNA-Free Kit (Thermo Fisher Scientific, Waltham, MA, USA), and RNasin^®^ Plus RNase Inhibitor (Promega, Madison, WI, USA) was added to HUVEC samples to prevent RNA degradation. RNA quality and quantity were assessed using the Agilent 2100 Bioanalyzer (Agilent Technologies, Santa Clara, CA, USA) and a NanoDrop spectrophotometer (Thermo Fisher Scientific, Waltham, MA, USA). A total of 500 ng of totRNA was used to prepare RNA libraries with the Stranded mRNA Prep, Ligation Kit (Illumina, San Diego, CA, USA), following the manufacturer’s instructions. Briefly, the mRNA fraction was purified via polyA capture, fragmented, and subjected to first-strand cDNA synthesis using reverse transcriptase and random primers. Second-strand synthesis was performed by incorporating dUTP instead of dTTP to ensure strand specificity. A- and T-bases were added to the fragment ends, index adapters were ligated, and cDNA was amplified via PCR. Library quality was validated using the Agilent Bioanalyzer (DNA 1000 Kit), and concentrations were measured with the Qubit™ dsDNA BR Assay Kit (Thermo Fisher Scientific, Waltham, MA, USA). Libraries were normalized, multiplexed, and sequenced on the Illumina NovaSeq 6000 system using the NovaSeq 6000 S1 Reagent Kit v1.5 (Illumina, San Diego, CA, USA).

BCL files were converted to FASTQ format and demultiplexed using bcl2fastq v2.20.0.422. Quality control of raw reads was performed with FastQC v0.11.9. Adapters and low-quality reads were trimmed using Cutadapt v2.8, and the processed reads were aligned to the Human Reference Genome GRCh38.99 using STAR v2.7, following index creation from the corresponding FASTA file. Read quantification was performed using FeatureCounts v2.0. Differential expression analysis was conducted using the DESeq2 package (version n. 1.36.0), which normalized raw count data, estimated biological variance, and identified differentially expressed genes between experimental conditions. Volcano plots were generated using the *ggplot2* R package (Version 4.0.0 to display differentially expressed genes (absolute fold change > 1.5 and adjusted *p* < 0.05). Functional enrichment analysis was performed with DAVID (https://davidbioinformatics.nih.gov/ accessed on 25 November 2025), and heatmaps were created using the p*heatmap* R package (Version 1.1.13).

### 2.9. Statistical Analysis

Data from experiments with at least three technical replicates per treatment were analyzed using GraphPad Prism™ software (Version 10.4.2) and reported as mean ± standard deviation (SD) or mean ± standard error of the mean (SEM). Differences between treatments were evaluated using one-way ANOVA, followed by Šídák’s multiple comparisons method, or using unpaired *t*-test with Welch’s correction. Statistical significance was defined as *p* < 0.05 (*), *p* < 0.01 (**), *p* < 0.001 (***), and *p* < 0.0001 (****).

## 3. Results

### 3.1. Dt Selectively Reduces Viability in TNBC Cells

**Figure 1:**
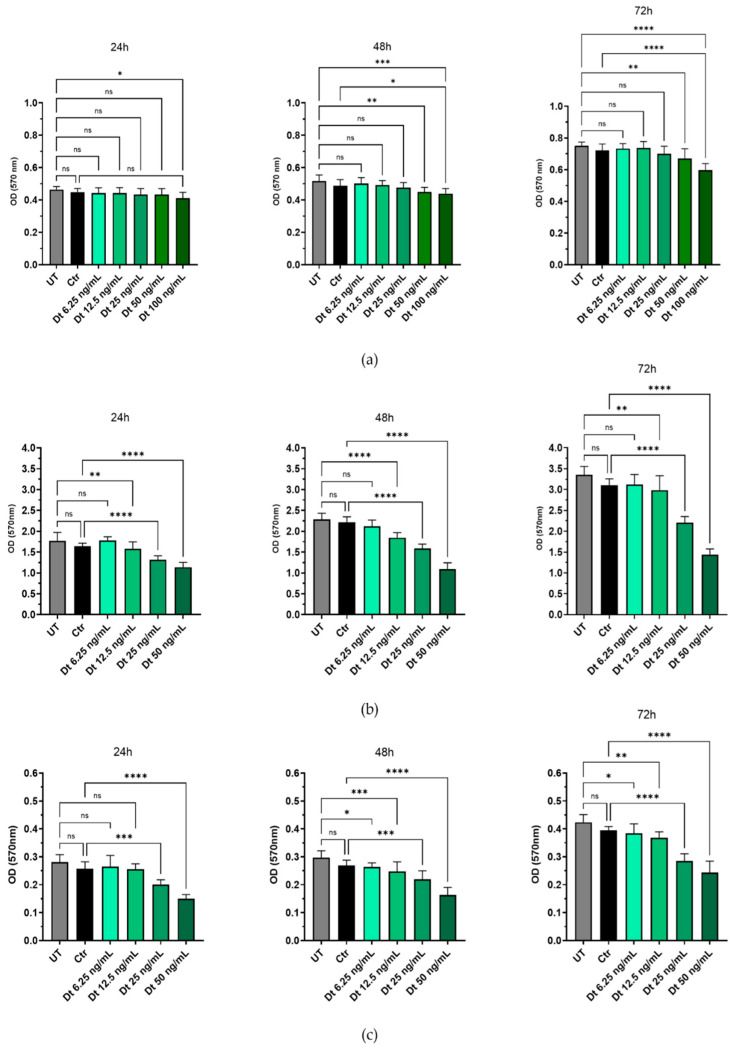
The effects of increasing concentrations of Dt on HUVECs and TNBC cell viability assessed using the MTT assay at different timepoints (24, 48, 72 h). Dose-curve effect or of its solvent only (Ctr) of Dt on HUVEC (**a**), MDA-MB-231 (**b**), and on BT-549 (**c**) cells. Data are presented as mean ± SD and analyzed using one-way ANOVA. Each experiment was performed in triplicate, with MTT OD measurements obtained from eight replicate wells per condition. UT = untreated cells, Ctr = control cells, Dt = diatoxanthin; ns = not significant. * *p* < 0.05, ** *p* < 0.01, *** *p* < 0.001, **** *p* < 0.0001.

Dt was preliminarily tested on HUVECs to determine a concentration that did not induce cytotoxicity in this cell type. Concentrations between 6.25 ng/mL and 25 ng/mL did not significantly affect HUVEC viability at 24, 48 or 72 h compared to UT. However, Dt at 50 ng/mL significantly reduced cell viability at 24 h, 48 h and 72 h, with a more pronounced effect observed at 100 ng/mL (*p* < 0.001, *p* < 0.001, *p* < 0.0001, respectively). No significant differences in viability were detected between cells treated with medium containing the solvent ethanol (Ctr) at the same dilution and UT at either timepoint, confirming that the observed effects were attributable to Dt (Figure 1a). Dt significantly reduced cell viability in MDA-MB-231 and BT-549 cells at concentrations ≥12.5 ng/mL, with maximal effects observed at 25 ng/mL (Figure 1b,c). These results underscore the potential of Dt’s selective anti-proliferative activity toward cancer cells.

**Figure 2:**
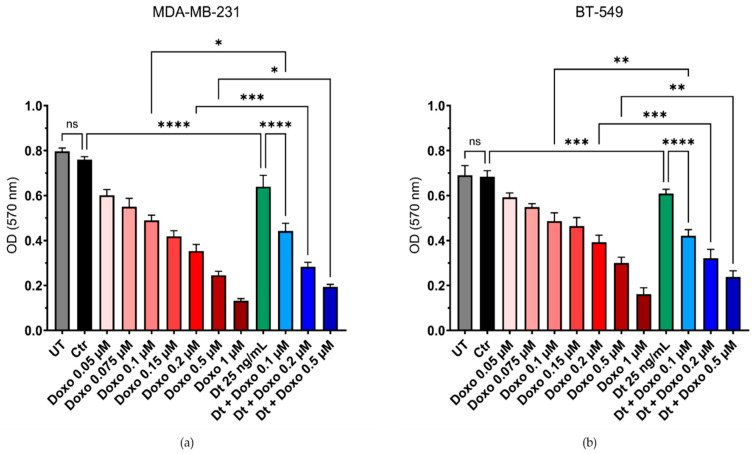
MTT assay using a combination of Doxo and Dt on TNBC cells. Viability of MDA-MB-231 (**a**) and BT-549 (**b**) cells after 48 and 72 h of treatment with increasing concentrations of Doxo and its combination with Dt (25 ng/mL). Data are presented as mean ± SD and analyzed using one-way ANOVA. Each experiment was performed in triplicate. UT = untreated cells; Ctr = control cells; Doxo = doxorubicin; Dt = diatoxanthin; ns = not significant. * *p* < 0.05, ** *p* < 0.01, *** *p* < 0.001, **** *p* < 0.0001.

The combination of Dt (25 ng/mL) with the chemotherapeutic agent Doxo was evaluated for potential additive effects against TNBC cell lines MDA-MB-231 and BT-549. Cell viability was assessed using the MTT assay following treatment with increasing concentrations of Doxo (0.05–1 μM) alone or in combination with Dt. Co-treatment with Dt enhanced the cytotoxic effects of Doxo in both TNBC cell lines. For MDA-MB-231 cells, combined treatment with Dt significantly reduced the cell viability compared to Doxo alone at 0.1 μM (* *p* < 0.05), 0.2 μM (*** *p* < 0.001), and 0.5 μM (* *p* < 0.05, Figure 2a). Similarly, for BT-549 cells, an enhanced effect was observed at 0.1 μM (** *p* < 0.01), 0.2 μM (*** *p* < 0.001), and 0.5 μM (** *p* < 0.01, Figure 2b).

### 3.2. Analysis of Dt and Doxo Effects on 3D-Tumor Spheroid Growth

**Figure 3:**
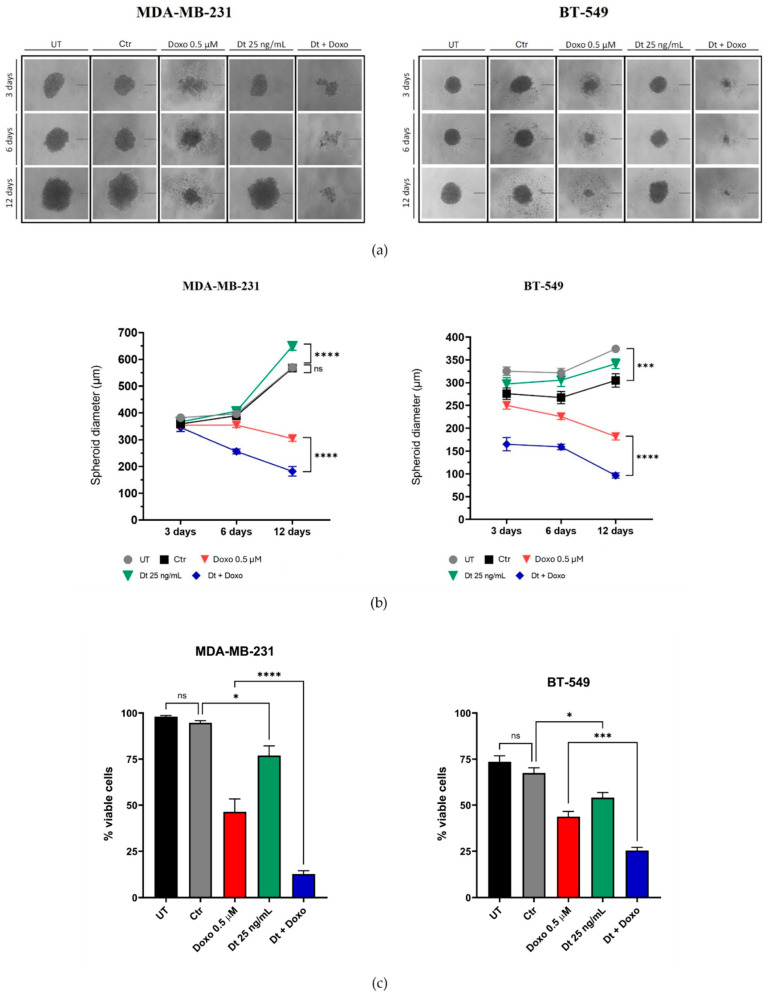
Effects of Dt, Doxo, and their combination on 3D-tumor spheroid growth in MDA-MB-231 and BT-549 cells. (**a**) Representative spheroid images at three time points (scale bar: 200 μm; four spheroids per condition). (**b**) Spheroid diameters across treatment conditions at each time point. (**c**) Crystal violet viability assay showing the percentage of viable cells at the end of the experiment relative to the initial cell count. Data are presented as mean ± SD, analyzed using one-way ANOVA. UT = untreated cells; Ctr = control cells; Doxo = doxorubicin; Dt = diatoxanthin; ns = not significant. * *p* < 0.05, *** *p* < 0.001, **** *p* < 0.0001.

The impact of Dt (25 ng/mL) and Doxo (0.5 μM) on the 3D-tumor spheroid growth of TNBC cell lines (MDA-MB-231 and BT-549) was assessed at 3, 6, and 12 days post-treatment. Dt alone did not significantly affect spheroid size, while Doxo reduced spheroid growth at all time points. Combined Dt and Doxo treatment demonstrated an enhanced effect, further reducing spheroid size and increasing morphological instability compared to Doxo alone (Figure 3a,b). Crystal violet assays performed at day 12 confirmed these findings, with Dt alone slightly reducing cell viability (*p* < 0.05), and the combined treatment showing a more pronounced reduction in viability (MDA-MB-231, *p* < 0.0001; BT-549, *p* < 0.001), indicating a potential enhancement effect (Figure 3c).

### 3.3. Dt Reduces TNBC Cell Migration

**Figure 4:**
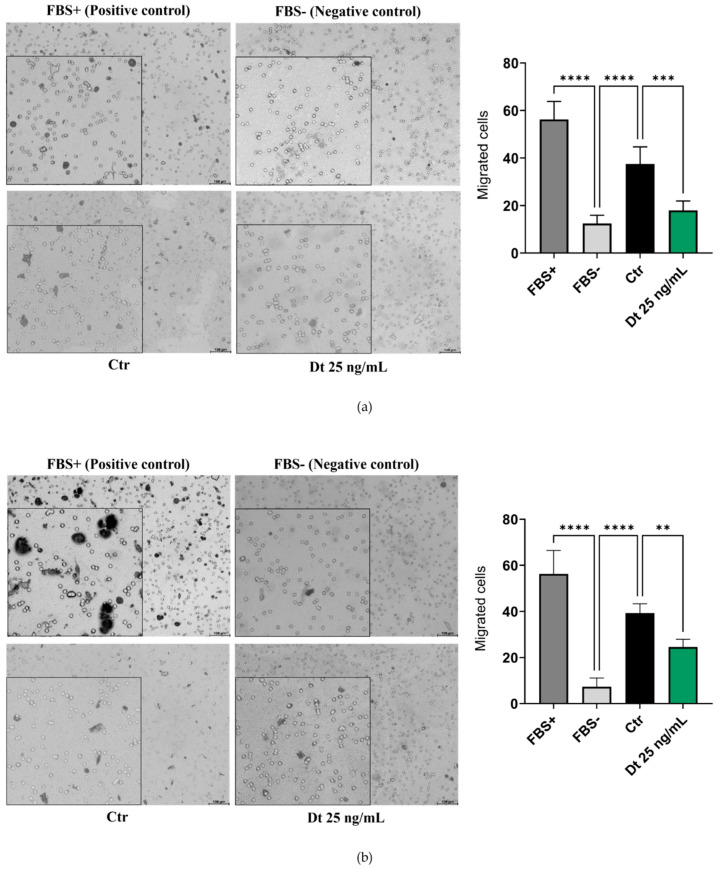
Transwell migration assay on TNBC cell lines. MDA-MB-231 (**a**) and BT-549 (**b**) TNBC cell lines treated with Dt. Migrated cells were quantified in three independent fields, and the values in the bar graphs represent the mean of three experimental replicates. Data are presented as mean ± SD, analyzed using one-way ANOVA. The experiment was repeated three times, each with three replicates. FBS = fetal bovine serum; Ctr = control cells; Dt = diatoxanthin; ns = not significant. ** *p* < 0.01, *** *p* < 0.001, **** *p* < 0.0001.

The results of the Transwell migration assay were comparable for both TNBC cell lines analyzed. Figure 4a,b illustrates a reduction in cell migration in Dt (25 ng/mL)-treated cells compared to the Ctr, and this reduction was confirmed as significant by statistical analyses.

**Figure 5:**
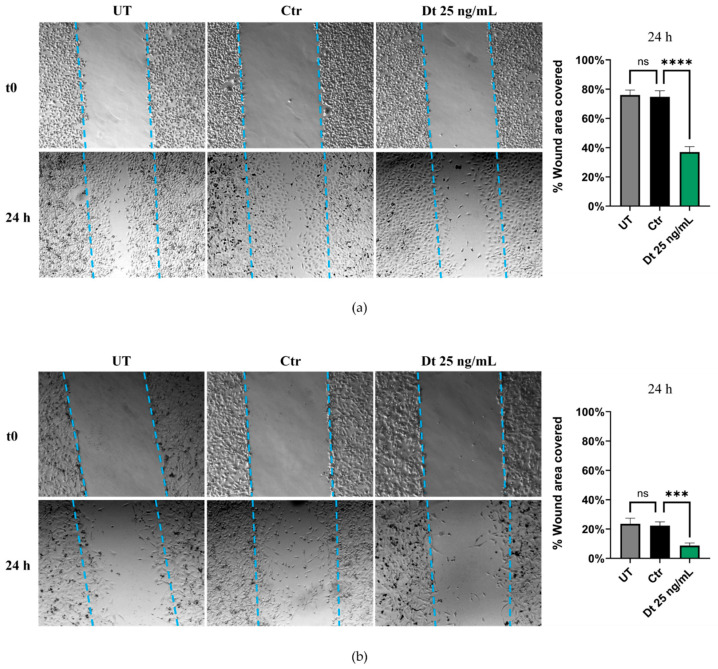
Wound healing scratch migration assay. Wound covered areas of MDA-MB-231 (**a**) and BT-549 (**b**) cell lines treated with Dt or vehicle only. The experiment was repeated three times, with each condition performed in triplicate. The bar graphs in the right-hand panels were generated for each replicate by measuring the area covered by migrated cells in four independent fields, 24 h post-scratch, for each experimental condition. UT = untreated; Ctr = control cells; Dt = diatoxanthin. ns = not significant. *** *p* < 0.001, **** *p* < 0.0001.

The wound healing assay was performed under three conditions: UT, Dt-treated cells, and vehicle control cells. Both TNBC cell lines exhibited a similar trend. After 24 h of treatment, Dt-treated cells showed a pronounced reduction in the wound-covered area compared to the control. This is depicted in the panels on the right in Figure 5a,b, which highlight a significant decrease in the percentage of wound area covered by Dt-treated cells relative to solvent control cells. These findings suggest a potential role for Dt in reducing the migratory capacity of these TNBC cell lines.

### 3.4. Effect of Dt on Gene Expression in the MDA-MB-231 and BT-549 TNBC Cell Lines

Gene expression profiling in the MDA-MB-231 and BT-549 TNBC cell lines was evaluated after 6 h of treatment with Dt (25 ng/mL).

**Figure 6:**
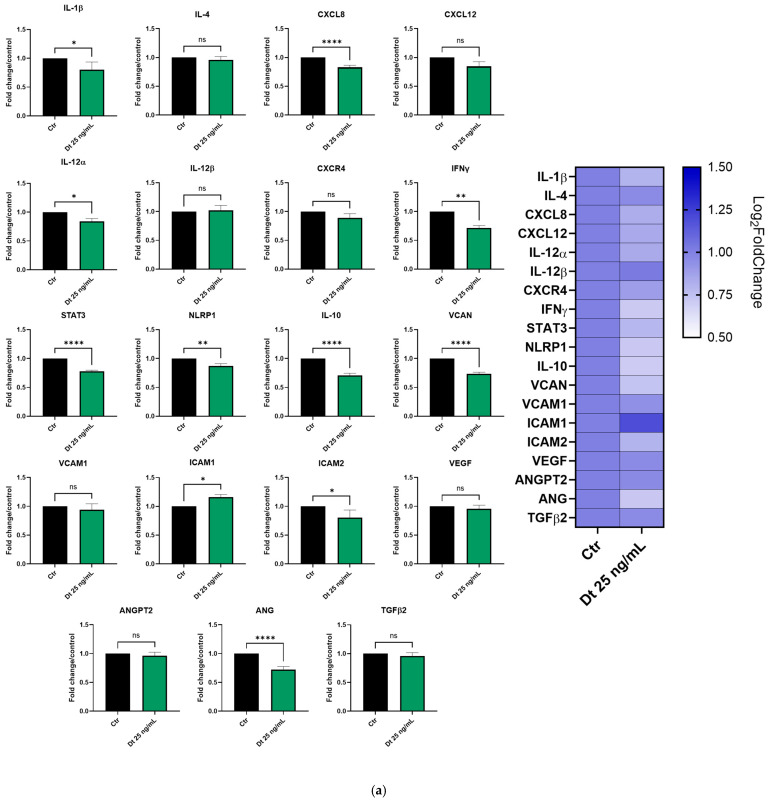

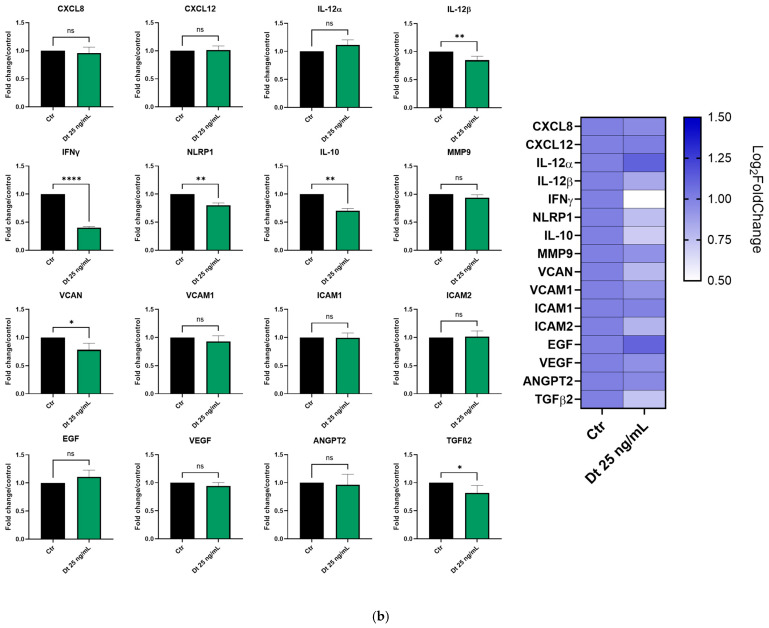
Gene expression profiling in the MDA-MB-231 (**a**) and BT-549 (**b**) TNBC cell lines treated with Dt for 6 h, analyzed by qPCR. Results are displayed as bar graphs for individual genes and summarized in a heatmap. Data represent relative mRNA expression normalized to β-actin and are presented as mean ± SD. Statistical analysis was performed using one-way ANOVA. Experiments were conducted in triplicate, with three replicates per condition. Ctr = control cells; Dt = diatoxanthin; ns = not significant. * *p* < 0.05, ** *p* < 0.01, **** *p* < 0.0001.

In MDA-MB-231 cells (Figure 6a), important pro-inflammatory genes such as *IL-1β*, *CXCL8*, *IL-12α*, *IFNγ*, and *STAT3* were significantly downregulated by Dt. Furthermore, although not statistically significant, the treatment with Dt slightly decreased the expression of other important inflammation mediators, such as *IL-4*, *CXCL-12* and *CXCR4*. In addition, Dt treatment showed an effect on angiogenesis-related genes (*VCAM*, *VCAN*, *VEGF*, *ANGPT2*, *ANG*) by downregulating their expression. Its effect on *ANG* and *VCAN* expression seems to be particularly evident.

In BT-549 cells (Figure 6b), Dt treatment led to a decrease in *IL-12β*, *IFN-γ*, *IL-10*, *VCAN*, and *TGF-β*, while its effect on the other tested genes appeared milder compared to the MDA-MB-231 cell line. Nevertheless, despite the slight not significant down modulation in some genes, an anti-inflammatory and anti-angiogenic trend appeared clear in the gene expression profile (Figure 6).

### 3.5. RNA-Seq Transcriptome Analysis in MDA-MB-231 Cells

**Figure 7:**
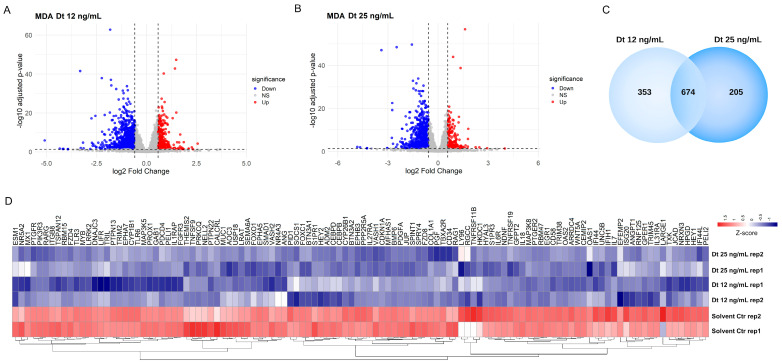
Heatmap of differentially expressed genes in MDA-MB-231 cells after treatment with Dt. (**A**) Volcano plot of differentially expressed genes in MDA-MB-231 cells treated with 12 ng/mL diatoxanthin versus solvent control. Upregulated genes are shown in red (logFC > 0.58, adj. *p* < 0.05), and downregulated genes are shown in blue (logFC < −0.58, adj. *p* < 0.05). (**B**) Volcano plot of differentially expressed genes in MDA-MB-231 cells treated with 25 ng/mL diatoxanthin versus solvent. Upregulated genes are shown in red (logFC > 0.58, adj. *p* < 0.05), and downregulated genes are shown in blue (logFC < −0.58, adj. *p* < 0.05). (**C**) Venn diagram showing the overlap of significantly downregulated genes in MDA-MB-231 cells treated with 12 ng/mL and 25 ng/mL diatoxanthin. (**D**) Heatmap of genes commonly downregulated at both diatoxanthin concentrations, showing significant changes in biological processes including blood vessel development, blood vessel morphogenesis, angiogenesis, regulation of cytokine production, cellular response to cytokine stimulus, cytokine signaling in the immune system, inflammatory response, and regulation of angiogenesis. Gene expression levels are represented as a gradient from red to blue, corresponding to positive and negative Z-scores, respectively.

The global transcriptomic changes induced by Dt treatment were analyzed by RNA sequencing. In both the Dt 12 ng/mL and Dt 25 ng/mL treatment conditions, more transcripts were downregulated than upregulated relative to the solvent-matched control (Figure 7A) and Dt 25 ng/mL (Figure 7B) treatments. Functional enrichment analysis using the 674 commonly downregulated genes (Figure 7C) revealed modulation of biological processes related with blood vessel development and morphogenesis, regulation of angiogenesis, cytokine signaling in the immune system, and inflammatory response. These genes include *ANGPT1*, *GPR37*, *ANG*, *QRICH2*, and *MUC1*, as well as IL-6 receptor (*IL-6R*) and *IL-7* (Figure 7D).

### 3.6. Secretome Analysis Using RayBiotech^®^ Human Inflammation Antibody Array and Human Angiogenesis Antibody Array

**Figure 8:**
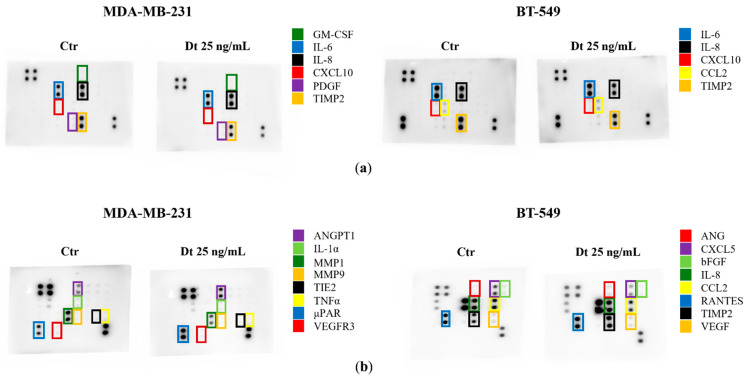
Secretome Analysis of TNBC Cell Lines treated with Dt using Inflammation and Angiogenesis-related antibody arrays. (**a**) Antibody array images obtained using RayBiotech^®^ human Inflammation antibody array C3 after 8 s of exposure. (**b**) Antibody array images obtained using RayBiotech^®^ human Angiogenesis antibody arrays C2 (MDA-MB-231) and C1 (BT-549) after 8 s of exposure. Ctr = control cells; Dt = diatoxanthin.

Secretome changes, particularly in inflammatory mediators and angiogenic factors, were evaluated in the MDA-MB-231 and BT-549 TNBC cell lines treated with Dt (25 ng/mL). Dt treatment showed a decrease in the amount of TIMP-2 protein released in the secretome of the MDA-MB-231, although its effect on other inflammatory mediators was relatively mild (Figure 8a). Notably, a more evident effect was clear in the analysis of the Angiogenesis Antibody Array, where Dt seemed to decrease the levels of MMP-1, MMP-9, and TNF-α (Figure 8b). In BT-549 cells, Dt did not show any effect in modulating inflammatory mediators released in the secretome (Figure 8a), while it caused a slight reduction in CXCL5, visible in the Angiogenesis Array (Figure 8b). These findings also suggest minor changes in angiogenesis inflammatory mediators at the protein level.

### 3.7. Effect of Dt on Gene Expression in HUVECs

**Figure 9:**
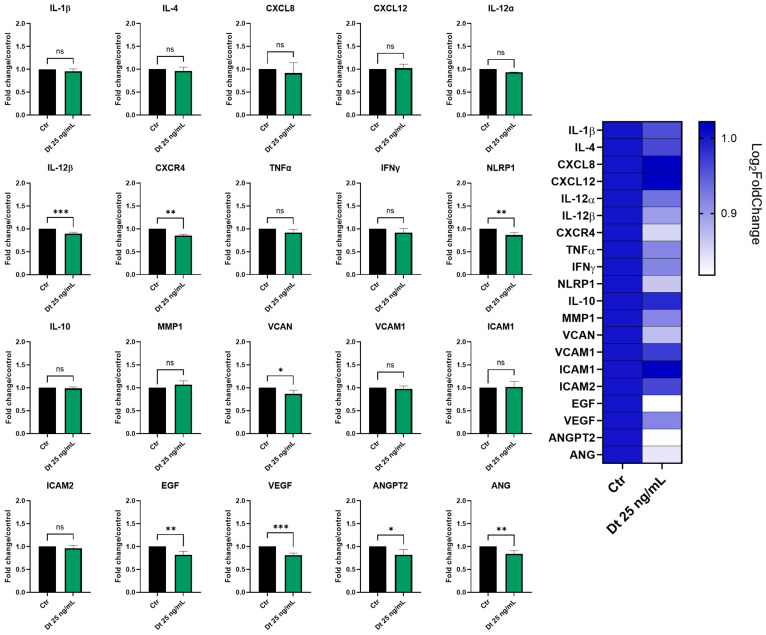
Inflammatory and angiogenesis gene expression analyzed by qPCR in TNF-α-stimulated HUVECs treated with Dt. Cells were treated with Dt (25 ng/mL) for 6 h and compared to control cells. Data are shown as mRNA relative expression, normalized to control, mean ± SEM, one-way ANOVA. The experiment was conducted in triplicate, with three replicates per condition. Ctr = control cells; Dt = diatoxanthin. ns = not significant. * *p* < 0.05, ** *p* < 0.01, *** *p* < 0.001.

The effects of Dt (25 ng/mL) on TNF-α-stimulated HUVEC gene expression were assessed by qPCR after 6 h of treatment. As shown in Figure 9, Dt caused a significant downregulation of some neo-angiogenesis-related genes, including *VEGF*, *EGF*, *VCAN*, *ANGPT2*, and *ANG*. Among the inflammation-associated genes, *TNF-α*, *IFNγ*, *CXCR4*, *NLRP1* and *IL-12β* showed a significant decrease in expression (Figure 9). *MMP1* was also decreased.

### 3.8. RNA-Seq Transcriptome Analysis in HUVECs

**Figure 10:**
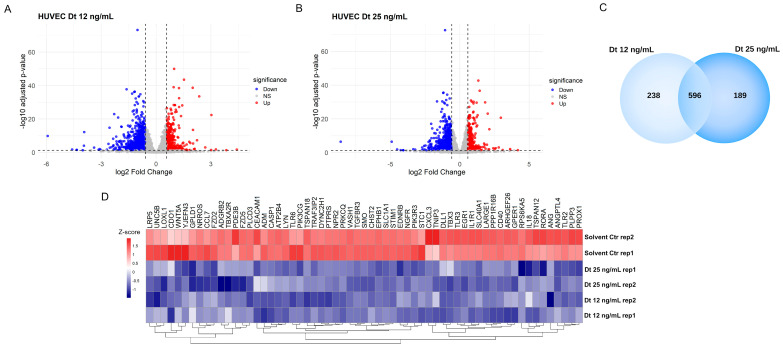
Differentially expressed genes in HUVECs after treatment with Dt. (**A**) Volcano plot of differentially expressed genes in HUVECs treated with 12 ng/mL diatoxanthin versus solvent. Up-regulated genes are shown in red (logFC > 0.58, adj. *p* < 0.05), and downregulated genes are shown in blue (logFC < −0.58, adj. *p* < 0.05). (**B**) Volcano plot of differentially expressed genes in HUVECs treated with 25 ng/mL diatoxanthin versus solvent. (**C**) Venn diagram of genes significantly downregulated (adjusted *p* < 0.05 and logFC < –0.58) in HUVECs treated with diatoxanthin at 12 ng/mL and 25 ng/mL, showing the overlap between the two dose conditions. (**D**) Heatmap of genes commonly downregulated at both diatoxanthin doses shows regulation of genes involved in vasculature development, inflammatory response, endothelium development, and regulation of angiogenesis. Gene expression levels are represented as a gradient from red to blue, corresponding to positive and negative Z-scores, respectively.

Functional enrichment analysis identified genes whose expression was significantly downregulated in HUVECs by Dt treatment at both 12 ng/mL and 25 ng/mL concentrations (Figure 10) compared to TNF-α-treated HUVECs, which is evidence that they participate in key pathways regulating vascular development, endothelial function, and angiogenesis. These findings suggest that Dt’s primary effect might be the suppression of TNF-α-induced pro-angiogenic transcription.

## 4. Discussion

The use of natural compounds in cancer therapy has garnered significant attention due to their diverse bioactive properties, lower toxicity profiles, and ability to target multiple hallmarks of cancer [4]. Among these, marine-derived compounds have emerged as a particularly promising class due to the unique chemical structures and bioactivities of molecules synthesized in marine environments [44,45,46]. Carotenoids such as xanthophylls have demonstrated potent antioxidant, anti-inflammatory, and anti-tumor properties, including activity as a ferroptosis inducer, with several showing promise in preclinical cancer models [5,12,21]. Dt, a xanthophyll pigment produced by diatoms, represents a novel marine compound with therapeutic potential [22]. Given the challenges of TNBC, such as limited treatment options, systemic toxicity of chemotherapy, and drug resistance [28,33], this cancer type represents an optimal model to study the chemopreventive potential of natural compounds like Dt and their ability to enhance chemotherapy efficacy [34]. Besides this, since there is a strong rationale that supports the exploring of Dt’s anticancer properties in other malignancies, the use of TNBC cell lines would deepen the knowledge about Dt’s role in cancer treatment.

Our findings are in favor of a selectivity of Dt in reducing TNBC cell viability (MDA-MB-231 and BT-549 cell lines) while sparing HUVECs. This selectivity is critical for minimizing off-target effects associated with conventional chemotherapies. The observed cytotoxic effects align with the role of Dt in inducing prostate cancer cell death [22] and a selective reduction in TNBC cell viability [21].

Notably, our findings suggest that when combined with Dt, the anti-proliferative effect of Doxo is enhanced, as in both TNBC cell lines, either proliferation in 2D culture or 3D-tumor spheroid growth was significantly suppressed. The 3D culture also showed increased morphological instability in the combined treatment. Interestingly, both Transwell migration and scratch/wound healing assays suggest that Dt significantly inhibits TNBC cell motility. Inhibition of cell migration is increasingly recognized as an important component of anti-tumor activity [47], particularly in highly aggressive cancers such as TNBC, where metastatic dissemination is the major determinant of patient prognosis [48]. The wound healing (scratch) assay is a well-established and reproducible method to assess two-dimensional collective cell migration in vitro, and is commonly used to determine whether anticancer agents can impair the ability of tumor cells to move and close a cell-free gap [49]. Therefore, compounds capable of impairing cell motility in scratch migration assays might potentially exert anti-metastatic effects, even when their cytotoxic activity is moderate. In this context, the significant reduction in TNBC cell migration observed following Dt treatment suggests that the compound may interfere with key pathways governing the invasive potential of TNBC cells.

Dt downregulated key inflammatory genes (*CXCL8/IL-8*, *IL-1β*, *IL-4*, *IL-12*, *STAT3*, *IFNγ*, *NLRP1*) as well as endothelial–tumor crosstalk genes (e.g., *VCAN*, *VCAM1*/*ICAM1*, *VEGF*/*VEGFA*, *ANG*/*ANGPT2*). These signatures are highly relevant in TNBC, where IL-6/JAK/STAT and chemokine networks sustain pro-angiogenic VEGF output and metastatic behavior.

Beyond these pathways, Dt also reduced cytokines linked to metastasis. IL-10, associated with TNBC bone metastasis, declined with Dt treatment, supporting an anti-metastatic effect [50]. Likewise, transforming growth factor-β, TGF-β, another pro-metastatic cytokine, was significantly decreased in BT-549 cells after Dt exposure, suggesting additional potential to curb metastatic processes [51]. Dt’s modulation of these axes could mitigate pro-tumor inflammation and dissemination [52]. Secretome analyses corroborated these findings: pro-tumorigenic and angiogenic proteins (TNF-α, CXCL5, CCL2) and matrix remodelers, such as MMPs, were reduced.

Transcriptomic profiling aligned with these results. In MDA-MB-231 cells, Dt downregulated genes involved in angiogenesis and tumor progression, including *ANGPT1*, *ANG*, *GPR37*, *QRICH2*, and *MUC1*, as well as *IL-6R* and *IL-7*. In TNF-α-stimulated HUVECs, Dt suppressed angiogenesis-related genes (*NOS3*, *DLL1*, *PPP1R16B*, *ADGRA2*, *NPR1*). At the protein level, Dt-treated HUVECs also showed decreased angiostatin, MMP-9, IL-2, and IL-4. Collectively, these data suggest that Dt exerts anti-angiogenic and anti-inflammatory effects in both tumor cells and the endothelial compartment of the tumor microenvironment, raising the prospect of limiting inflammation-driven angiogenesis and tumor progression.

Taken together, the gene expression and secretome results consistently show a downregulation of inflammation- and angiogenesis-related genes and secreted mediators that are functionally linked to metastatic progression. Specifically, the downregulation of IL-6R and signal transducer and activator of transcription 3 (STAT3), together with TGF-β, has been consistently associated with a reduced migratory phenotype of TNBC cells [53,54,55]. Nevertheless, further investigation of the Dt-modulated molecular pathways regulating cell migration is warranted, given their relevance in the context of the highly metastatic TNBC subtype.

Compared to another study that reported very low IC_50_ values (≈6 ng/mL in MDA-MB-231 cells), highlighting the potency of Dt [21], the present study focused on a therapeutically relevant concentration range (12.5–25 ng/mL) suitable for functional and combination experiments. In addition, our analysis included two TNBC cell lines (MDA-MB-231 and BT-549) as well as HUVECs, thereby extending the evaluation across multiple TNBC models. Furthermore, in this study, Dt was found to reduce tumor cell migration and chemotaxis and limit 3D spheroid growth, providing functional information that was not examined in the previous report.

Dt’s potential multifaceted anti-tumor activities, ranging from selective cytotoxicity and inflammation modulation to angiogenesis inhibition and migration suppression, pose it as a promising chemopreventing candidate in TNBC. The observed enhancement in the reduction in TNBC cell proliferation induced by Doxo when associated to Dt suggests Dt as an effective supplement, enhancing chemotherapy efficacy while possibly minimizing adverse effects. The observation that Dt further reduces TNBC cell proliferation when combined with Doxo suggests its potential as an effective agent, capable of enhancing chemotherapy efficacy while minimizing adverse effects. This finding would be crucial, but further investigation is needed to shed light on the potential adjuvant role of Dt.

Moreover, Dt’s natural origin and established antioxidant properties make it a potential candidate for integration into dietary or pharmacological strategies for cancer prevention and management, following the path laid out by its sibling molecule, fucoxanthin, already commercially available as a dietary supplement [56].

Hence, its potential use within pharmacological strategies for cancer prevention and management would be facilitated. Although this study provides in vitro insights of Dt’s potential against TNBC cells, further investigations are needed for possible clinical applications. Dt’s impact on endothelial cells within the tumor microenvironment represents an interesting field of research. Investigating Dt’s effects across other TNBC subtypes, cancer models and tumor microenvironment models, may reveal its broader therapeutic relevance and potential for combinatorial strategies with other chemotherapeutic agents or immunotherapies.

## 5. Conclusions

The antioxidant xanthophyll Dt likely displays significant potential as a natural agent against TNBC and its anti-proliferative activities enhanced those of Doxo. The ability of this natural antioxidant to selectively target cancer cells, modulate inflammation and angiogenesis, and decrease the migration capacity, with gene expression modulation demonstrated by qPCR and RNA-Seq analysis, offers a novel, multifaceted approach to address the challenges associated with aggressive TNBC subtypes. Future research should focus on translating these findings into preclinical and clinical contexts, paving the way for innovative treatment strategies in oncology.

## Data Availability

The original contributions presented in this study are included in the article and Supplementary Materials. Further inquiries can be directed to the corresponding author.

## Supplementary Materials

The following supporting information can be downloaded at: https://www.mdpi.com/article/10.3390/antiox15020205/s1, Figure S1: (A) 2D formula and (B) 3D structure of diatoxanthin. National Center for Biotechnology Information (2026). PubChem Compound Summary for CID 6440986, Diatoxanthin. https://pubchem.ncbi.nlm.nih.gov/compound/Diatoxanthin (accessed 16 January 2026). Table S1: List and sequences of primers used for qPCR analyses.
