# Supplementary material for: The Algal Antioxidant Carotenoid Diatoxanthin as a Modulator of Inflammation and Angiogenesis in Triple-Negative Breast Cancer Cells
Source: Antioxidants (Basel). 2026 Feb 4;15(2):205. doi: 10.3390/antiox15020205 (PMC12938071; doi:10.3390/antiox15020205)
Supplement: Supplementary file 1 [file antioxidants-15-00205-s001.zip › antioxidants-4065566-supplementary.pdf]

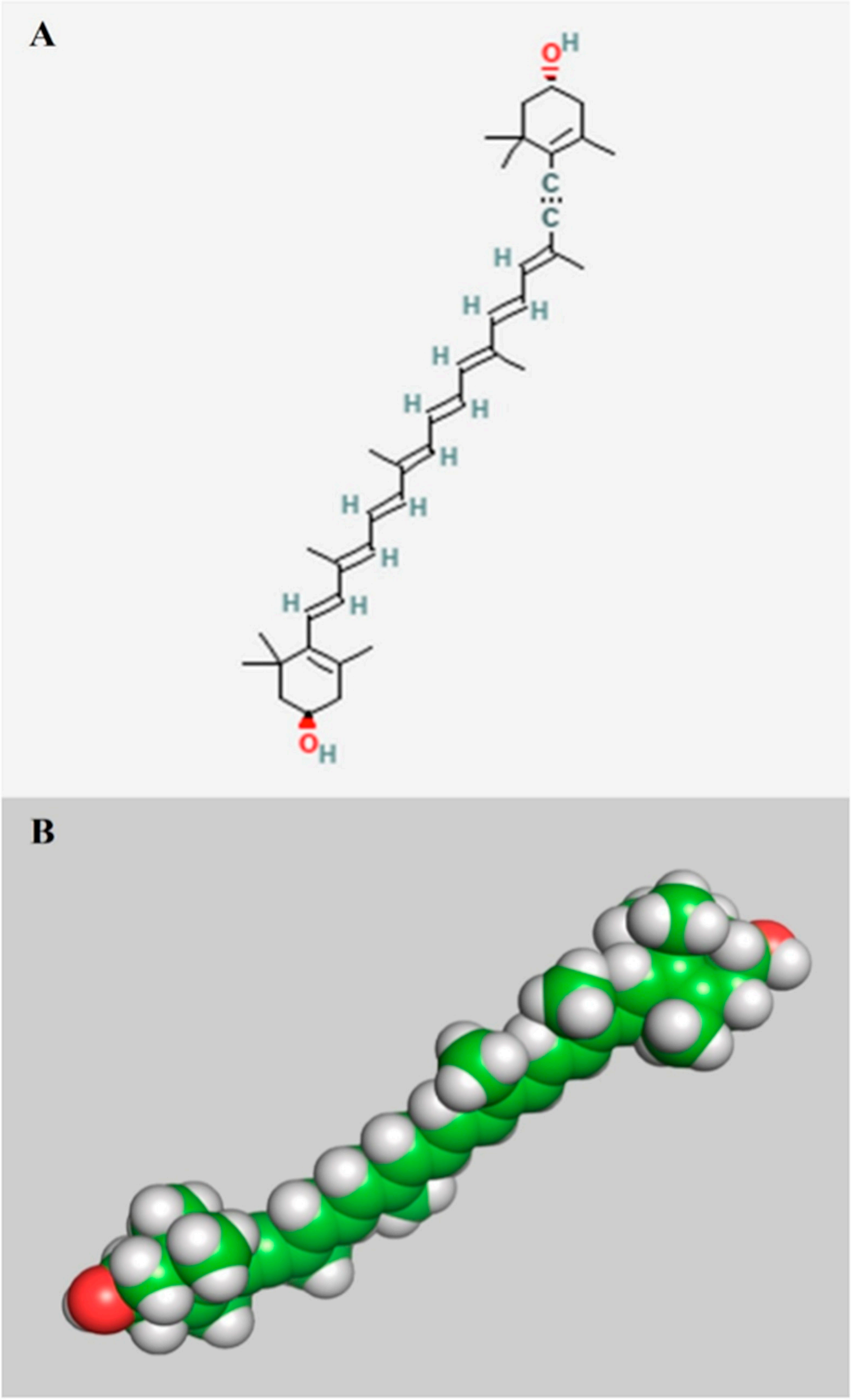

Figure S1. (A) 2D formula and (B) 3D structure of diatoxanthin. National Center for Biotechnology Information (2026). PubChem Compound Summary for CID 6440986, Diatoxanthin. https://pubchem.ncbi.nlm.nih.gov/compound/Diatoxanthin (accessed 16 January 2026).

Table S1. Sequences of primers used for qPCR analyses. For = Forward; Rev = Reverse.

Gene	Primer	Sequence
hANG	For	5' - TGGCAACAAGCGCAGCATCAAG - 3'
	Rev	5' - GCAAGTGGTGACCTGGAAAGAAG - 3'
hANGPT2	For	5' - ATTCAGCGACGTGAGGATGGCA - 3'
	Rev	5' - GCACATAGCGTTGCTGATTAGTC - 3'
hCXCL12	For	5' - CTCAACACTCCAAACTGTGCCC - 3'
	Rev	5' - CTCCAGGTACTCCTGAATCCAC - 3'
hCXCL8	For	5' - GAGAGTGATTGAGAGTGGACCAC - 3'
	Rev	5' - CACAACCCTCTGCACCCAGTTT - 3'
hCXCR4	For	5' - CTCCTCTTTGTCATCACGCTTCC - 3'
	Rev	5' - GGATGAGGACACTGCTGTAGAG - 3'
hEGF	For	5' - TGCGATGCCAAGCAGTCTGTGA - 3'
	Rev	5' - GCATAGCCCAATCTGAGAACCAC - 3'
hICAM1	For	5' - AGCGGCTGACGTGTGCAGTAAT - 3'
	Rev	5' - TCTGAGACCTCTGGCTTCGTCA - 3'
hICAM2	For	5' - ATGACACGGTCCTCCAATGCCA - 3'
	Rev	5' - GCACTCAATGGTGAAGGACTTGC - 3'
hIL10	For	5' - TCTCCGAGATGCCTTCAGCAGA - 3'
	Rev	5' - TCAGACAAGGCTTGGCAACCCA - 3'
hIL12α	For	5' - TGCCTTCACCACTCCCAAAACC - 3'
	Rev	5' - CAATCTCTTCAGAAGTGCAAGGG - 3'
hIL12β	For	5' - GACATTCTGCGTTCAGGTCCAG - 3'
	Rev	5' - CATTTTTGCGGCAGATGACCGTG - 3'
hIL1β	For	5' - CCACAGACCTTCCAGGAGAATG - 3'
	Rev	5' - GTGCAGTTCAGTGATCGTACAGG - 3'
hIL4	For	5' - CCGTAACAGACATCTTTGCTGCC - 3'
	Rev	5' - GAGTGTCCTTCTCATGGTGGCT - 3'
hINFγ	For	5' - CTAATTATTCGGTAACTGACTTGA - 3'
	Rev	5' - ACAGTTCAGCCATCACTTGGA - 3'
hMMP1	For	5' - ATGAAGCAGCCCAGATGTGGAG - 3'
	Rev	5' - TGGTCCACATCTGCTCTTGGCA - 3'
hMMP2	For	5' - AGCGAGTGGATGCCGCCTTTAA - 3'
	Rev	5' - CATTCCAGGCATCTGCGATGAG - 3'
hMMP9	For	5' - GCCACTACTGTGCCTTTGAGTC - 3'
	Rev	5' - CCCTCAGAGAATCGCCAGTACT - 3'
hNFKB	For	5' - GCAGCACTACTTCTTGACCACC - 3'
	Rev	5' - TCTGCTCCTGAGCATTGACGTC - 3'
hNLRP1	For	5' - ATTGAGGGCAGGCAGCACAGAT - 3'
	Rev	5' - CTCCTTCAGGTTTCTGGTGACC - 3'
hSTAT3	For	5' - CTTTGAGACCGAGGTGTATCACC - 3'
	Rev	5' - GGTCAGCATGTTGTACCACAGG - 3'
hTGFβ2	For	5' - AAGAAGCGTGCTTTGGATGCGG - 3'
	Rev	5' - ATGCTCCAGCACAGAAGTTGGC - 3'
hTNFα_1	For	5' - CCTCTCTCCCCTGGAAAGGA - 3'
	Rev	5' - CAAAGTGCAGCAGGCAGAAG - 3'
hVCAM1	For	5' - GATTCTGTGCCCACAGTAAGGC - 3'
	Rev	5' - TGGTCACAGAGCCACCTTCTTG - 3'
hVCAN	For	5' - TTGGACCTCAGGCGCTTTCTAC - 3'
	Rev	5' - GGATGACCAATTACACTCAAATCAC - 3'
hVEGF	For	5' - TTGCCTTGCTGCTCTACCTCCA - 3'
	Rev	5' - GATGGCAGTAGCTGCGCTGATA - 3'
